# Complete nucleotide sequence of a virus associated with rusty mottle disease of sweet cherry (*Prunus avium*)

**DOI:** 10.1007/s00705-013-1668-9

**Published:** 2013-03-23

**Authors:** D. V. Villamor, K. L. Druffel, K. C. Eastwell

**Affiliations:** 1 Department of Plant Pathology, Irrigated Agriculture Research and Extension Center, Washington State University, Prosser, WA 99350 USA; 2 Department of Plant Pathology, Washington State University, Pullman, WA 99164 USA; 3 Present Address: Department of Plant Pathology, University of Florida-GCREC, 14625, County Rd 672, Wimauma, FL 33598 USA

## Abstract

Cherry rusty mottle is a disease of sweet cherries first described in 1940 in western North America. Because of the graft-transmissible nature of the disease, a viral nature of the disease was assumed. Here, the complete genomic nucleotide sequences of virus isolates from two trees expressing cherry rusty mottle disease symptoms are characterized; the virus is designated cherry rusty mottle associated virus (CRMaV). The biological and molecular characteristics of this virus in comparison to those of cherry necrotic rusty mottle virus (CNRMV) and cherry green ring mottle virus (CGRMV) are described. CRMaV was subsequently detected in additional sweet cherry trees expressing symptoms of cherry rusty mottle disease.

**Electronic supplementary material:**

The online version of this article (doi:10.1007/s00705-013-1668-9) contains supplementary material, which is available to authorized users.

## Introduction

Cherry rusty mottle disease (CRMD) is a graft-transmissible disease of sweet cherry (*Prunus avium*) first described in 1940 in Washington State, USA [1]. Affected trees of sensitive cultivars exhibit chlorotic mottling on the basal leaves that abscise prematurely, while the remaining leaves develop bright yellow or red mottling [2]. Many aspects of CRMD symptomatology resemble those of cherry necrotic rusty mottle disease (CNRMD) [3]. However, CNRMD and CRMD are distinguished by distinct symptoms induced in different woody hosts. On specific hosts, CNRMD is associated with large angular necrotic leaf spots, whereas CRMD-affected leaves develop yellow mottle symptoms with a bronze overtone. Cherry green ring mottle virus (CGRMV) also occurs frequently in *P. avium*, where it does not induce acute symptoms.

The complete nucleotide (nt) sequences of CGRMV and the virus associated with CNRMD (cherry necrotic rusty mottle virus [CNRMV]) were reported previously [4, 5]. CNRMV and CGRMV are currently unassigned members of the family *Betaflexiviridae* [6, 7]. Reverse transcription polymerase chain reaction (RT-PCR) amplicons with sequence similarity to CGRMV were reported in association with CRMD [8], and a complete viral genomic sequence was reported from a tree exhibiting CRMD. It was later described to consist of 8,401 nucleotides (nt), sharing 70% and 68% nt sequence identity with CNRMV and CGRMV, respectively [9 as cited in 10]. At the time of this writing, this sequence has yet to be deposited in a public database.

To better understand virus-like agents of sweet cherry, the complete genomic sequence of a virus associated with CRMD was determined. Since Koch’s postulates for the etiology of CRMD have not yet been established, the virus sequenced in this study is designated cherry rusty mottle associated virus (CRMaV).

**Fig. 1:**
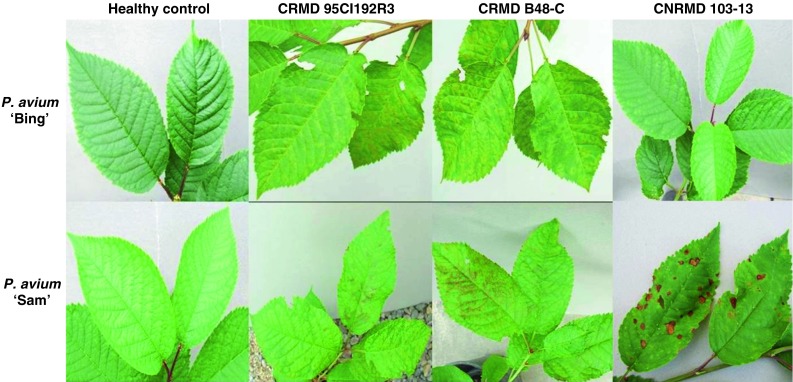
Comparison of symptoms induced on woody indicators *Prunus avium* ‘Bing’ and ‘Sam’ 90 days after graft inoculation of a bark patch from cherry rusty mottle disease (CRMD) sources 95CI192R3 and B48-C and cherry necrotic rusty mottle disease (CNRMD) source 103-13

The reference sources for CRMD and CNRMD were maintained in *P. avium* ‘Bing’ and ‘Sam’, respectively, in screenhouses of the Clean Plant Center Northwest (CPCNW), located at Washington State University, Prosser, WA. Graft inoculation of indicator trees with bark chips from CRMD reference sources 95CI192R3 or B48-C resulted in the induction of typical rusty mottle symptoms consisting of chlorotic yellow leaf mottling on ‘Bing’ and chlorotic yellow mottle overlaid with reddish brown mottling on ‘Sam’ (Fig. 1). In contrast, inoculation with CNRMD reference source 103-13 did not induce any acute symptoms on ‘Bing’ but resulted in necrotic lesions on ‘Sam’ reported to be characteristic of CNRMD. Two additional CRMD sources, 8079-2 and 8099-3, collected from commercial sweet cherry orchards in Washington State and one CGRMD reference source 02F23rD from the CPCNW were included in the analysis of the viral coat protein gene but were not used in the biological woody indexing.

The presence of common viruses and viroids of cherry was investigated by RT-PCR (Supplementary table S1 for primers). A one-tube RT-PCR system was used (SuperScript® III One-Step RT-PCR System with Platinum® *Taq*; Life Technologies Corporation, Carlsbad, CA) following the manufacturer’s recommendation with the addition of 14 μg non-acetylated bovine serum albumin per 25 μl reaction. Prune dwarf virus was present in both CRMD sources 95CI192R3, B48-C and in CNRMD source 103-13. CRMD 95CI192R3 was also infected with both Prunus necrotic ringspot virus and cherry virus A. RT-PCR tests for the presence of other cherry viruses, viroids and two bacteria were negative in all CRMD and CNRMD reference samples.

Preliminary analysis of double-stranded RNA from affected trees yielded sequences closely related to CNRMV and CGRMV (unpublished); therefore, a forward primer (Fovea2: CTCA-TTC-ACA-TAG-CTT-AGGT) was designed from conserved regions located upstream of the coat protein region of full genomic sequences of CGRMV (AF017780; AJ291761) and CNRMV (AF237816; EU188438, and EU188439). First-strand cDNA synthesis was initiated from total RNA with FRSTD primer (TATG-ACA-CGC-GTC-GAC-TAGC(T)_17_) [11] and reverse transcriptase (SuperScript™ III; Life Technologies). PCR amplification utilized primer pair Fovea2 and AdPr (TATG-ACA-CGC-GTC-GAC-TAGC) with DNA polymerase (Platinum® *Taq* DNA Polymerase; Life Technologies). The 1.1-kbp products were ligated into pCR-2.1 (Life Technologies) and three clones of each amplification reaction were sequenced in both directions (ELIM Biopharmaceuticals, Inc., Hayward, CA).

The sequence at the 5’-terminus was obtained by designing primers (NGRM 6161R: GCAT-CCA-ATT-CTT-TTT-CGG-ACA-TTAT, and NGRM 2996R: GCAT-GCC-AGA-AGC-AAT-CCC-CATC) from the conserved regions in the multiple sequence alignments of CGRMV and CNRMV. NGRM 6161R was used for first-strand cDNA synthesis, and NGRM 2996R was employed in 5’ rapid amplification of cDNA ends (5’-RACE) using a commercial kit (GeneRacer®: Life Technologies). Five clones of the resulting amplification products of 3 kbp were sequenced.

Primers were designed from the sequences obtained above to amplify the full-length genomes in a two-step RT-PCR reaction. First-strand cDNA was initiated with a 3’-terminal, CRMaV-specific primer (CRM 95CI192R3 3’-end R1: GCA-TTG-CAA-ACT-AAA-GGA-AAA-T-ATAT); cDNA was used for PCR with the 5’-terminal primer (CRM 5’-end F1: GAAA-ACAA-ACAG-ACCA-AAAC-TAGC) and the 3’-terminal primer CRM 95CI192R3 3’-end R1 with DNA polymerase (PrimeSTAR**®** HS DNA Polymerase: Takara Bio, Inc., Otsu, Shiga, Japan). An A-overhang was added to the product with DNA polymerase (Taq DNA Polymerase, recombinant; Life Technologies), and the resulting product of 8.4 kbp was ligated into pCR**®-**XL-TOPO (Life Technologies). Three different clones from a single RT-PCR reaction were selected for sequencing by primer walking. Assembly of contigs and pairwise alignment of sequences were performed with BioEdit [12]. Multiple sequence alignments of the coat protein amino acid (aa) sequences were performed with ClustalW [13], and phylogenetic trees were constructed using the neighbor-joining algorithm with 1000 bootstrap replicates of MEGA, version 5 [14].

**Fig. 2:**
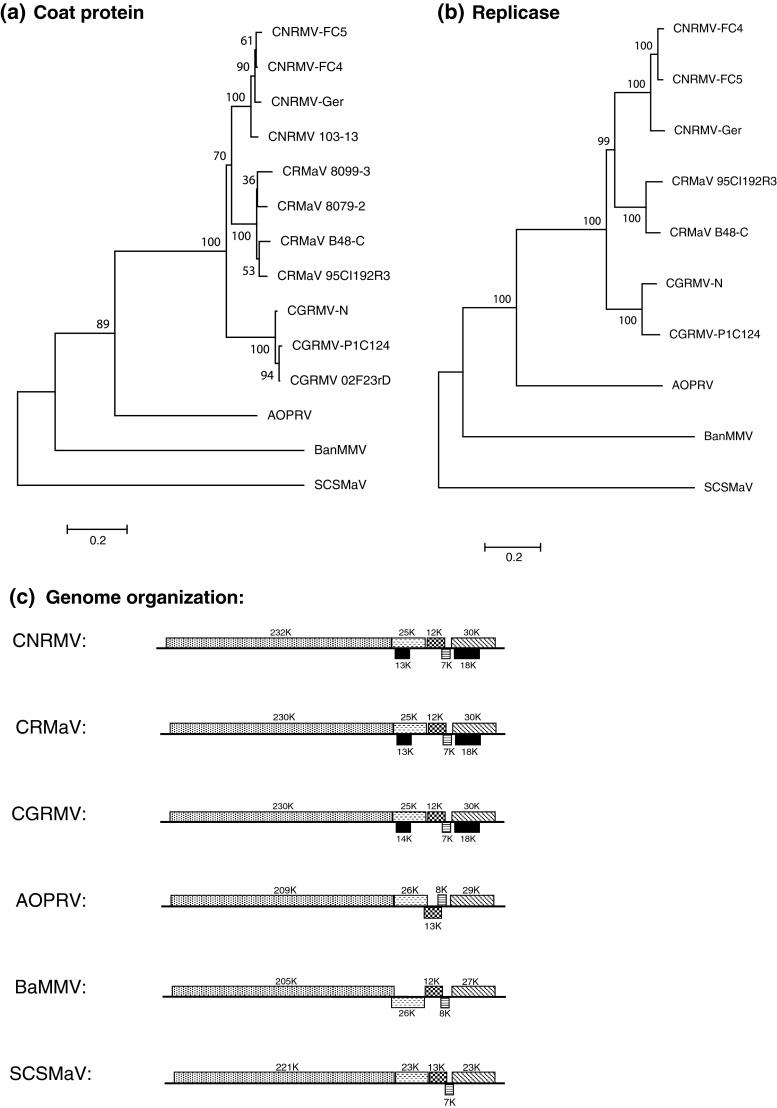
Phylogenetic analysis (**a** and **b**) and genome organization comparison (**c**) of cherry rusty mottle associated virus (CRMaV) and cherry necrotic rusty mottle virus (CNRMV), two cherry green ring mottle virus (CGRMV) isolates, African oil palm ringspot virus (AOPRV), banana mild mosaic virus (BanMMV) and sugarcane striate mosaic-associated virus (SCSMaV). The alignment was derived from the ClustalW alignment of coat protein (**a**) and replicase (**b**) amino acid sequences, and the unrooted tree was generated using the neighbor-joining algorithm of the MEGA5 program package (see text for details)

Two virus-like RNA sequences were obtained from reference trees affected with CRMD. The complete nucleotide (nt) sequences from CRMD reference trees 95CI192R3 and B48-C consist of 8,397 and 8,398 nt, respectively, excluding the poly A-tail (GenBank accessions KC218926 and KC218927, respectively). The viruses represented by these sequences are designated cherry rusty mottle associated virus (CRMaV), and the sequences were compared with those of known isolates of CGRMV and CNRMV, including three other unassigned members of the family *Betaflexiviridae* (Table 1). Nucleotide sequences of CRMaV isolates revealed a closer relationship to CNRMV than to CGRMV; this is further supported by analysis of the aa sequences of the putative CP and replicase coding regions. The predicted CP aa sequences from the four CRMaV source trees formed a distinct clade (Fig. 2a). Sequences obtained from CNRMD 103-13 and CGRMD 02F23rD (GenBank accessions KC218930 and KC218931, respectively) clustered with CNRMV and CGRMV, respectively, as anticipated.

**Table 1:** Percent nucleotide (nt) and amino acid (aa) sequence identities of cherry rusty mottle associated virus (CRMaV) 95CI192R3 to three other CRMaV sources, cherry green ring mottle virus (CGRMV), cherry necrotic rusty mottle virus (CNRMV), African oil palm ringspot virus (AOPRV), banana mild mosaic virus (BanMMV) and sugarcane striate mosaic-associated virus (SCSMaV)

**Virus** ^**a**^	**Genomic sequence (nt)**	**5’-UTR (nt)**	**ORF1 (aa)**	**Triple gene block**			**ORF5 (aa)**	**ORF2a (aa)**	**ORF5a (aa)**	**3’-UTR (nt)**
				**ORF2 (aa)**	**ORF3 (aa)**	**ORF4 (aa)**				
CRMaV B48-C	83	94	90	95	86	90	94	91	89	91
CRMaV 8079-2	nd^b^	nd	nd	nd	nd	nd	94	nd	82	94
CRMaV 8099-3	nd	nd	nd	nd	nd	nd	92	nd	80	88
CNRMV 103-13	nd	nd	nd	nd	nd	nd	80	nd	42	74
CNRMV-FC4	72	71	72	82	54	60	80	73	48	75
CNRMV-FC5	72	69	72	82	54	63	80	75	50	74
CNRMV-Ger	72	70	73	82	55	60	80	72	45	74
CGRMV 02F23rD	nd	nd	nd	nd	nd	nd	73	nd	42	67
CGRMV-N	70	77	70	78	55	57	73	57	43	68
CGRMV-P1C124	70	77	70	77	55	58	72	56	41	67
AOPRV	58	22	41	50	33	30	41	n/a^c^	n/a	19
BanMMV	52	27	28	35	32	31	28	n/a	n/a	17
SCSMaV	55	30	29	23	19	19	20	n/a	n/a	18

^a^Sequences obtained from GenBank: CNRMV–FC4, EU188438; CNRMV–FC5, EU188439; CNRMV–Ger, AF237816; CGRMV–N, AF017780; CGRMV–P1C124, AJ291761; AOPRV, NC_012519; BanMMV, NC_002729; SCSMaV, NC_003870

^b^nd, not determined

^c^n/a, not applicable; no corresponding ORF is present

Overall, the genome organization of CRMaV 95CI192R3 and B48-C is similar to that of CGRMV and CNRMV, with five presumed ORFs plus two ORFs nested within ORFs 2 and 5 and designated ORF 2a and 5a, respectively (Fig. 2b). The 5’-UTR of CRMaV 95CI192R3 and B48-C are 94% identical to each other, consisting of 115 nt (Table 1); the pentamer GAAAA present in CGRMV [4, 18], CNRMV [5, 15] and most potexviruses [16] is located at nt 1 to 5 in CRMaV. The 3’-UTRs of CRMaV 95CI192R3, B48-C, 8079-2 (GenBank accession KC218928) and 8099-3 (GenBank accession KC218929) consist of 192, 193, 192 and 191 nt, respectively, and contain the conserved hexanucleotide ACUUAA. This motif is found in potex- and carlaviruses [17] and CGRMV [4, 18] but not CNRMV; it may have a role in viral RNA synthesis.

ORF1 of CRMaV encodes a protein with a relative molecular mass (M_r_) of 230 kDa containing the conserved domains for viral methyltransferase (Met), helicase (Hel) and RNA-dependent RNA polymerase (RdRp) associated with the replicases of plant viruses; a papain-like cysteine protease (P-Pro) that processes the replicase polyprotein; an AlkB homologue; and an ovarian tumor (OTU)-like cysteine protease. These domains are also found in the replicase regions of CGRMV and CNRMV.

ORFs 2 to 4 are analogous to the ‘potex-like’ cell-to-cell-movement-associated triple gene block (TGB) proteins [19]. ORF2 (TGBp1) is a viral RNA helicase of superfamily I containing the conserved NTP-binding motif GxxGxGKS/T. ORF3 (TGBp2) and ORF4 (TGBp3) encode polypeptides predicted to be transmembrane proteins containing a block of hydrophobic sequences and a conserved central region [19].

ORF5 encodes the putative CP, consisting of 269 aa with a calculated M_r_ of 30 kDa. The CP contains the conserved core motif present in potex- and carlavirus coat proteins, which is hypothesized to be involved in viral RNA–coat protein interaction [20]. This motif is also found in CNRMV and CGRMV coat proteins. Two other putative ORFs, ORF2a and ORF5a, have the potential to encode proteins consisting of 119 and 157 aa with a calculated M_r_ of 13 and 18 kDa, respectively. The existence of these putative ORFs nested within ORF2 and ORF5 is a unique feature found in CGRMV, CNRMV and CRMaV, but the expression of these ORFs has yet to be demonstrated. A database search using the default parameters of the *tblastx* program failed to identify proteins with significant similarity to the putative proteins encoded by these two ORFs.

Phylogenetic analysis of sequences of the large replication proteins (ORF1) of members of the family *Flexiviridae* led to the division of the family into two families, *Alphaflexiviridae* and *Betaflexiviridae* [21]. Members of the *Alphaflexiviridae* have a potex-like replication protein with a molecular mass < 195 kDa, whereas the corresponding proteins in members of the *Betaflexiviridae* are > 195 kDa, and termed “carlavirus-like replicase”. Since the replication protein (ORF1) of CRMaV is 230 kDa and shares a high degree of nt and aa sequence identity with CGRMV and CNRMV across all ORFs, it is evident that it is a member of the family *Betaflexiviridae.* The comparative values of aa sequences of replication proteins and CP between these viruses are above 45%, which is the minimum criterion for inclusion of the viruses within a single genus.

The proposed criterion for species demarcation in the family *Betaflexiviridae* is that members of distinct species have less than 72% nt sequence identity or 80% aa sequence identity in the whole CP or replication protein gene [6]. This criterion creates incongruence when applied to CNRMV, CGRMV and CRMaV. These viruses would be considered members of distinct species when the nt coding sequences of the replicase (data not shown) or the aa sequences of the replicase (ORF1) are compared. However, they would be considered isolates of the same virus when CP aa sequences are compared; CNRMV has 80% aa CP sequence identity to CRMaV 95CI192R3 (Table 1). At the nt level, all pairwise sequence comparisons in the CP region between four CRMaV isolates with either four CNRMV or three CGRMV isolates resulted in values of 72% nt or greater (data not shown). Since the creation of the family *Betaflexiviridae* from the now defunct “Flexiviridae” was based on the phylogenetic reassessment of the replicase coding region, the assignment of CNRMV, CGRMV and CRMaV to three separate virus species is favored. This assessment is supported by examination of the biological data. The distinction between CNRMV, CGRMV and CRMaV is exemplified by distinctly different symptoms induced on different *P. avium* cultivars. As additional virus sequences are reported, the species demarcation criteria for the family *Betaflexiviridae* must be revised to address contradictory interpretations when CP versus replicase coding regions products are considered.

The results of this study reveal a newly identified virus, CRMaV, which is closely related to but distinct from CGRMV and CNRMV, both of which are unclassified members of the family *Betaflexiviridae*. The process of genetic recombination could explain the relationships between the CP and replicase sequences of these three viruses. However, preliminary analyses using the Recombination Detection Program version 3 (RDP3) [22] for the occurrence of putative recombination events in the CP and replicase coding regions as well as full genomic sequences of these viruses did not reveal potential breakpoints for recombination (data not shown). Nevertheless, the potential for genetic recombination can not be overlooked, especially in situations where mixed infections of these viruses occur in a single tree. The close relationships among CRMaV, CNRMV and CGRMV isolates characterized in this study may signify that these three viruses are in the process of further speciation from a common ancestor, similar to that proposed for Plantago asiatica mosaic virus and tulip virus X, members of the genus *Potexvirus* [23].

## Electronic supplementary material

Below is the link to the electronic supplementary material.

Supplementary material 1 (DOC 62 kb)
