# Supplementary material for: Complete nucleotide sequence of a virus associated with rusty mottle disease of sweet cherry (Prunus avium)
Source: Arch Virol. 2013 Mar 23;158(8):1805–10. doi: 10.1007/s00705-013-1668-9 (PMC3723981; doi:10.1007/s00705-013-1668-9)
Supplement: Supplementary file 1 — Supplementary material 1 (DOC 62 kb) [file 705_2013_1668_MOESM1_ESM.doc]

| **Pathogen and primer name** | **Primer sequences** | **Annealing temp (^0^C)** | **Amplicon size (bp)** | **Reference** |
| --- | --- | --- | --- | --- |
|  | | | | |
| Genus *Ilarvirus, American plum line pattern* *virus* (APLPV): | | | | |
| APLPV 196 | GGTGCCCGTTTAATTCAGG | 55 | 224 | Eastwell, K.C., unpublished data |
| APLPV c420 | TATTGCTGCCTCACAAGTGG |  |  |  |
|  | | | | |
| Genu*s Ilarvirus, Prune dwarf virus* (PDV): | | | | |
| PDV 3 | CCCTCCTGCTGGTTTTGTTA | 60 | 176 | Rampitsch *et al.,* 1995 |
| PDV 5 | CACGGACTTTCATGGTGTAA |  |  |  |
|  | | | | |
| Genus *Ilarvirus, Prunus necrotic ringspot virus* (PNRSV): | | | | |
| PNRSV 16 | ATATTGGCAGGTACAGAAGG | 54 | 998 | Vaskova *et al.*, 2001 |
| PNRSV 17 | TTCGGAGAAATTCGAGTGTGC |  |  |  |
|  | | | | |
| Genus *Trichovirus, Apple chlorotic leafspot virus* (ACLSV): | | | | |
| ACLSV J3 | AGTCTGTAAAAGCCGGTTC | 58 | 450 | Spiegel *et al.*, 2006 |
| ACLSV J4 | CCTTCATGGAAAGACAGG |  |  |  |
|  | | | | |
| Genus *Trichovirus, Cherry mottle leaf virus* (CMLV): | | | | |
| PWD 6055F | TTAGCTTTGCTGAGGCTGTACCGA | 50 | 430 | Mekuria, T.M. and Eastwell, K.C. unpublished data |
| PWD 6848R | ACGTCCCTTGGATTGCAATGTTGG |  |  |  |
|  | | | | |
| Genus *Nepovirus, Cherry leafroll virus* (CLRV): | | | | |
| CLRV 44 | GACTGCAATCAGTTCCATGC | 55 | 215 | Eastwell, K.C., unpublished data |
| CLRV 259c | CCTAGCCAACGCTACCTACC |  |  |  |
|  | | | | |
| Genus *Nepovirus, Cherry raspleaf virus* (CRLV): | | | | |
| CRLV JQ3D3FF | GCCAGTTTCTCCAGTGAACC | 58 | 546 | James *et al.,* 2001 |
| CRLV 3185c | CACTAGGAAAGCTAAAACGA |  |  | Eastwell, K.C., unpublished data |
|  |  |  |  |  |

**Supplemental table 1.** List of primers used in PCR for the detection of other sweet cherry viruses and other pathogens in CRMD isolates 95CI192R3 and B48-C and CNRMD isolate 103-13.

| **Pathogen and primer name** | **Primer sequences** | **Annealing temp (^0^C)** | **Amplicon size (bp)** | **Reference** |
| --- | --- | --- | --- | --- |
|  | | | | |
| Genus *Capillovirus, Cherry virus A* (CVA): | | | | |
| CVA 4480 | ACTGGAGAATTCTGCACCT | 52 | 252 | Eastwell and Bernardy, 1998 |
| CVA c4732 | CTGGCTTCTTGACTATCCA |
|  | | | | |
| Family *Closteroviridae* (unassigned member), *Little cherry virus* 1 (LChV-1): | | | | |
| LCUW 7090 | GGTTGTCCTCGGTTGATTAC | 47 | 299 | Bajet *et al.,* 2008 |
| LCUW c7389 | GGCTTGGTTCCATACATCTC |
|  | | | | |
| Family *Closteroviridae*, Genus *Ampelovirus, Little cherry virus* 2 (LChV-2): | | | | |
| LC26R | GCAGTACGTTCGATAAGAG | 52 | 409 | Eastwell and Bernardy, 1996 |
| LC26L | AACCACTTGATAGTGTCCT |
|  | | | | |
| Genus *Hostuviroid*, *Hop stunt viroid* (HSVd): | | | | |
| HSVd 1 | GCCCCGGGGCTCCTTTCTCAGGTAGAG | 60 | 300 | Kusano *et al.,* 1997 |
| HSVd 2 | GGCAACTCTTCTCAGAATCC |
|  | | | | |
| Genus *Pelamoviroid*, *Peach latent mosaic viroid,* (PLMVd): | | | | |
| PLMVd 113 | TGCAGTGCTCCGAATAGG | 62 | 337 | Loreti *et al.,* 1999 |
| PLMVd 114 | GTTCCCGATAGAAAGGCTAAG |
|  | | | | |
| *Xyllela fastidiosa*: | | | | |
| Xyl RST 31 | GCGTTAATTTTCGAAGTGATTCGATTG | 55 | 733 | Minsavage *et al.,* 1994 |
| Xyl RST 33 | CACCATTCGTATCCCGGTG |
|  | | | | |
| Phytoplasma: | | | | |
| Phyt 399mod | GCCGCGTGAACGATGAATTA | 55 | 1300 | Skrzeczkowski *et al.*, 2001 |
| Phyt 1694 | ATCAGGCGTGTGCTCTAACC |
|  |  |  |  |  |
|  |  |  |  |  |
